# Primary Choriocarcinoma in Two Male Patients: A Case Report

**DOI:** 10.1002/ccr3.70903

**Published:** 2025-09-21

**Authors:** Abraam Rezkalla, Islam Rajab, Nagihan Orhun, Sarah Saife, Yasmin Rjoub

**Affiliations:** ^1^ Internal Medicine Resident Saint Joseph University Medical Center Paterson New Jersey USA; ^2^ Department of Medicine, Faculty of Medicine and Health Sciences An‐Najah National University Nablus Palestine; ^3^ Department of Medicine, Faculty of Medicine Palestine Polytechnic University Hebron Palestine

**Keywords:** chemotherapy, choriocarcinoma, extragonadal tumor, human chorionic gonadotropin, male germ cell tumor, metastasis, testicular cancer

## Abstract

Primary choriocarcinoma in males is an uncommon and aggressive subtype of germ cell tumors characterized by an early rise in human chorionic gonadotropin (hCG). While testicular cancers are the most common type of solid cancers among young males, almost half of the primary choriocarcinomas diagnosed have metastasized to lymph nodes, lungs, or liver at the time of presentation. Here we present two cases describing two middle‐aged men diagnosed with advanced malignancy of primary choriocarcinoma.

**Summary**

- Primary choriocarcinoma in males is a rare and highly aggressive malignancy often presenting with metastases at diagnosis. Early recognition, diagnosis via elevated human chorionic gonadotropin (hCG) levels, and aggressive treatment are crucial for improving survival, underscoring the need for heightened clinical suspicion and prompt intervention.

## 1 Introduction

Choriocarcinoma, particularly in the male population, is a rare but aggressive form of cancer that has both poor diagnostic and prognostic factors due to atypical presenting symptoms. In men, the primary tumor site is often the testes; however, extragonadal tumors can develop in the mediastinum, retroperitoneum, lungs, brain, or gastrointestinal system. It can be found in midline locations of the body, including the pineal gland, retroperitoneum, or mediastinum. Rare cases may be present in the lung and the gastrointestinal tract [1]. Being a rare testicular cancer, it is mainly composed of trophoblastic tissue (cytotrophoblasts and syncytiotrophoblasts). Trophoblasts function to assist in the implantation and placental development by secreting human chorionic gonadotropin (hCG); hence, why hCG is used as a tumor marker to identify choriocarcinoma [2].

Symptoms of choriocarcinoma may be mild, including scrotal swelling and a palpable testicular mass. However, patients may present with shortness of breath, abdominal pain, seizures, headaches, etc., tailored to where the disease has metastasized [3]. Patients with advanced metastatic disease may develop choriocarcinoma syndrome, which is noted to be hemorrhage from the metastatic regions and is associated with high levels of mortality. Overall, the prognostic factors and clinical features of primary choriocarcinoma are not well understood [4].

Choriocarcinoma is the rarest yet most aggressive form of germ cell tumor in males from 15 to 35 years old. Oftentimes, when diagnosed, these patients already have evidence of metastatic disease due to their propensity for hematogenous spread [5].

## 2 Case Presentation

### 2.1 Case 1

**FIGURE 1:**
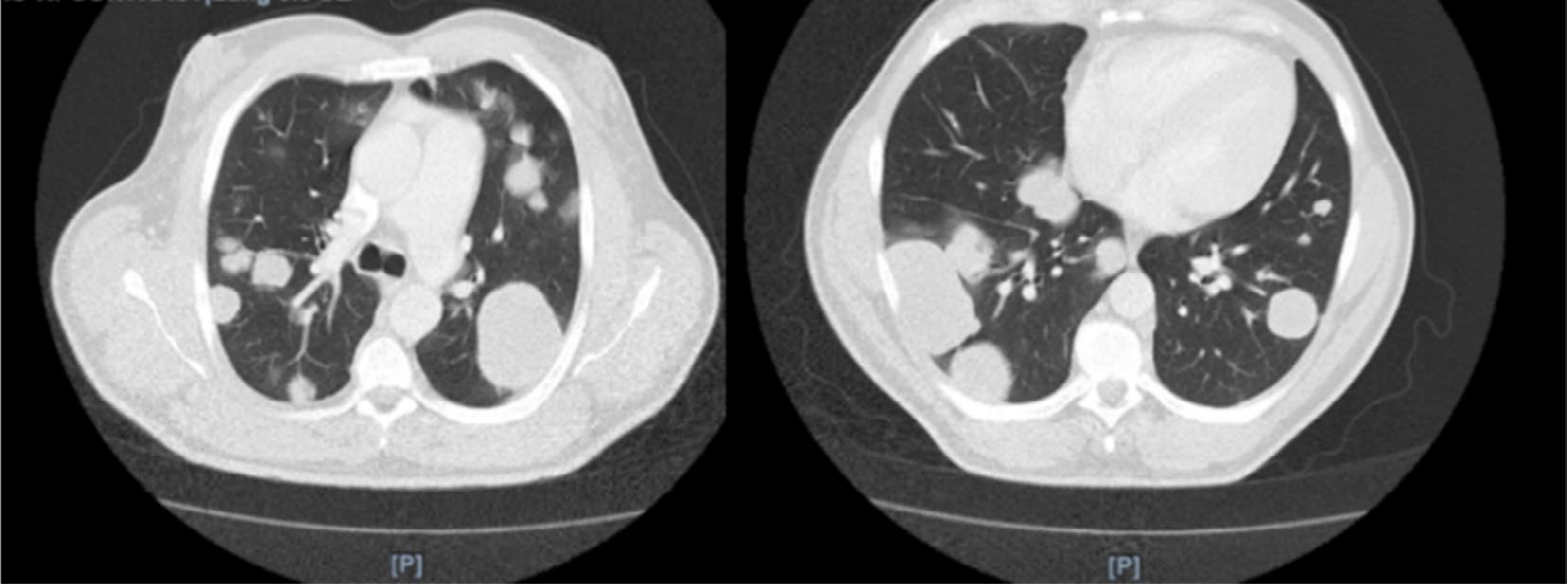
CT chest, abdomen pelvis with contrast. Numerous bilateral pulmonary masses with the largest measuring 6.8 cm in the right lower lobe and 6.2 cm in the left upper lobe. These findings are most likely secondary to metastatic disease.

**FIGURE 2:**
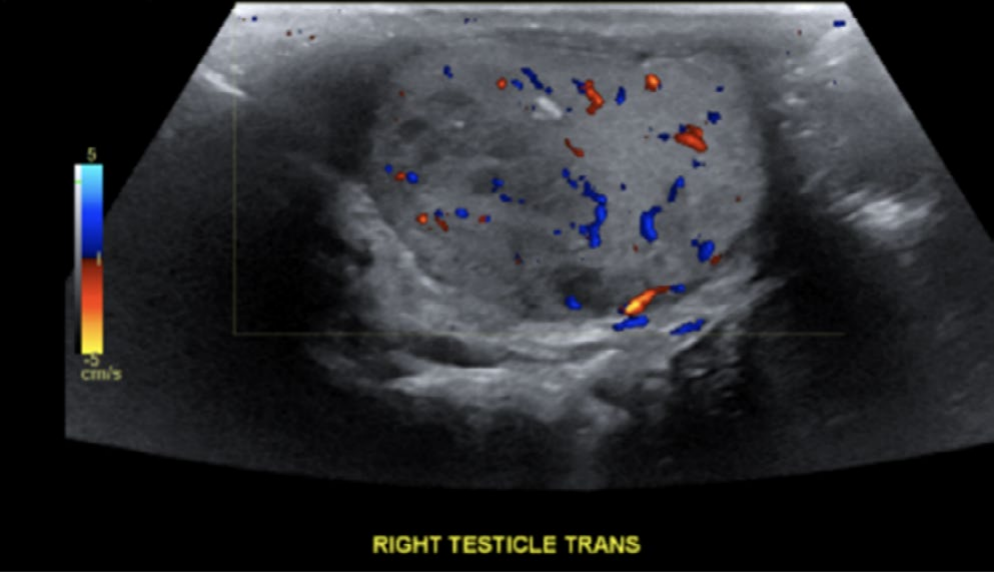
Scrotal ultrasound. Color Doppler and spectral waveform analysis utilized. Heterogeneous right testicular mass measuring 4.2 cm.

A 47‐year‐old male presented to the emergency department with worsening abdominal pain for 1 month. Associated symptoms included a 20‐pound weight loss, decreased appetite, early satiety, night sweats, and constipation. A computed tomography (CT) scan of the chest, abdomen, and pelvis without contrast revealed numerous pulmonary masses, mediastinal and hilar lymphadenopathy, suggesting metastatic disease (Figure 1). Ultrasound of the scrotum showed a 4.2 cm right testicular mass and a left varicocele (Figure 2). Magnetic resonance imaging (MRI) of the brain demonstrated an enhancing mass in the right occipital lobe with edema, likely representing metastasis. Laboratory findings revealed an elevated hCG level of > 200,000 mIU/mL, alpha‐fetoprotein (AFP) of 369 ng/mL, and carcinoembryonic antigen (CEA) of 1.1 ng/mL.

### 2.2 Case 2

**FIGURE 3:**
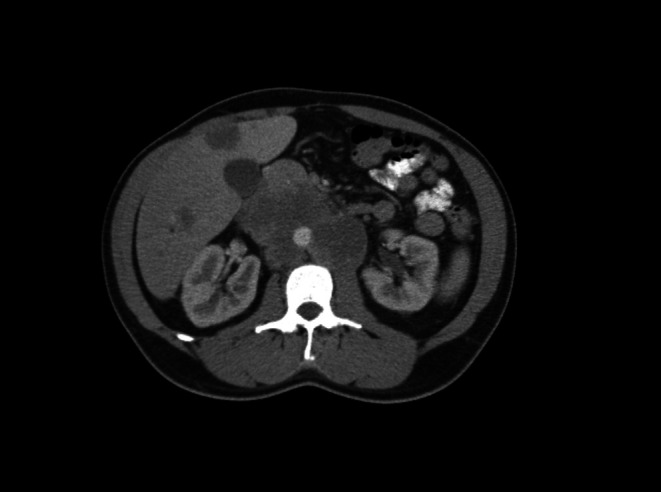
Multiple hepatic lesions with peripheral enhancement present, raising suspicions of metastatic disease.

A 44‐year‐old male with a history of alcohol use disorder presented to the emergency department after collapsing on the street. He reported 20 episodes of vomiting, diffuse abdominal pain (10/10 intensity), dizziness, and bloody vomit. He was hypotensive and diaphoretic. A focused assessment with sonography for trauma (FAST) revealed fluid in the right upper quadrant. CT angiography showed bilateral lung masses, hepatic lesions, a large hematoma anterior to the left liver lobe, and para‐aortic and aortocaval masses with peripheral enhancement (Figure 3). An ultrasound of the scrotum identified a complex 4.9 x 5 x 6 cm cystic/solid mass. Laboratory findings included hCG levels > 130,000 mIU/mL and lactate dehydrogenase (LDH) of 581 units/L.

## 3 Methods (Differential Diagnosis, Investigation, and Management)

For both cases, the initial differential diagnoses included testicular germ cell tumors such as seminoma, nonseminoma, embryonal carcinoma, yolk sac tumor, or teratoma. Other possibilities included metastatic carcinoma of unknown primary origin and other retroperitoneal or mediastinal malignancies.

Investigations performed included imaging studies, such as CT scans for detection of metastases, MRI to assess central nervous system involvement, and scrotal ultrasound to confirm testicular involvement. Laboratory markers, including elevated hCG levels, confirmed the diagnosis of choriocarcinoma, while AFP and CEA levels helped rule out other tumor types.

We did not perform a biopsy due to the patient's unstable condition and the clinical picture, along with elevated hCG levels, strongly suggesting choriocarcinoma.

## 4 Management

In the first case, the patient was started on an aggressive chemotherapy regimen, including bleomycin, etoposide, and cisplatin (BEP). Supportive care focused on symptom management and nutritional support. In the second case, the patient underwent an emergent exploratory laparotomy for hemoperitoneum and control of liver hemorrhage. Interventional radiology performed embolization of the bleeding liver tumor, and chemotherapy with BEP was initiated postoperatively.

## 5 Outcome and Follow‐Up

### 5.1 Case 1

The patient showed a partial response to chemotherapy, with a significant reduction in hCG levels. Follow‐up imaging demonstrated a decreased size of the pulmonary and brain metastases. He continues to receive treatment and undergo regular monitoring of tumor markers and imaging to assess disease progression.

### 5.2 Case 2

The patient stabilized postoperatively, with improved clinical symptoms and reduced hCG levels. Chemotherapy was ongoing at the time of reporting. Imaging showed decreased size of hepatic and pulmonary lesions. Regular follow‐up included monitoring hCG levels and repeat imaging to evaluate treatment efficacy.

Both cases highlight the importance of early diagnosis, comprehensive imaging, and aggressive multidisciplinary management in improving outcomes for patients with primary choriocarcinoma.

## 6 Discussion

Choriocarcinoma in extragonadal locations such as midline locations of the body, including the pineal gland, retroperitoneum, or mediastinum, is predominantly found in younger patients under the age of 34, while choriocarcinoma of parenchymal organs is most typical in older patients. Patients younger than 34 years of age are also found to have more favorable health outcomes, suggesting age is a prognostic factor [1]. Poor response to chemotherapy, metastasis to the brain, symptoms of hemoptysis, and a high burden of disease are also associated with poorer outcomes [2]. Due to the rare and complex nature of the disease, urgent referral to more specialized centers is essential for survival [3].

The disease is extremely rare, especially in men [6]. Male testicular choriocarcinoma accounts for less than 1% (0.19%) of testicular germ cell tumors, and its annual incidence is [2, 3, 4]/10 million, accounting for only 0.01% ~ 0.02% of male malignant tumors [7].

hCG is a key factor as it is used in both the diagnosis and prognosis of choriocarcinoma and is directly proportional to the estimated tumor burden. Levels of B‐hCG > 1000 mIntlUnit/mL are diagnostic, and this patient presented with levels > 130,000 mIntlUnit/mL [8]. With treatment, levels of hCG should be monitored over time to assess the success of the treatment.

Early treatment of choriocarcinoma is crucial to survival. Treatment includes orchidectomy, chemotherapy, radiotherapy, and lymph node dissection of the retroperitoneal nodes. Chemotherapy regimens may consist of BEP, etoposide and cisplatin (EP), and vinblastine or etoposide with isophosphamide and cisplatin (VIP) [9].

Though cisplatin‐oriented chemotherapy is the gold standard treatment of solid testicular germ cell tumors, there are numerous cases that demonstrate that treatment with chemotherapy alone is often ineffective [4]. In these patients, there have been some successes in monitoring levels of programmed death ligand 1 (PD‐L1) and supplementing chemotherapy with pembrolizumab [4]. Regularly measuring levels of PD‐L1 could be used as a prognostic factor in many patients to determine the progression of their disease, as well as the efficacy of the treatment regimen.

For limitations as with all case reports, the findings may not be generalizable to all settings, but they contribute valuable insights into the presentation and management of a rare and aggressive malignancy.

These cases highlight the difficulties associated with the diagnosis and early treatment of primary choriocarcinoma. In both cases, our patient presented with acute and nonspecific symptoms that led to proper imaging and investigation and eventual diagnosis.

## 7 Conclusion

Primary choriocarcinoma in men is a rare, aggressive malignancy with a poor prognosis. Due to its nonspecific symptoms and frequent presentation with metastatic disease, diagnosis is often delayed. These two cases highlight the necessity for high clinical suspicion, early imaging, and prompt initiation of combination chemotherapy. Improving diagnostic accuracy and facilitating early referral to specialized centers are critical for improving outcomes.

## Author Contributions

**Abraam Rezkalla:** conceptualization, project administration, writing – original draft, writing – review and editing. **Islam Rajab:** investigation, methodology, supervision. **Nagihan Orhun:** investigation, resources, validation, writing – original draft. **Sarah Saife:** writing – original draft, writing – review and editing. **Yasmin Rjoub:** writing – original draft, writing – review and editing.

## Ethics Statement

This study was conducted in accordance with the ethical standards of the institution. Approval was obtained from the relevant ethics committee.

## Consent

Written informed consent was obtained from both patients to publish this case report and accompanying images.

## Conflicts of Interest

The authors declare no conflicts of interest.

## Data Availability

The authors have nothing to report.
